# Case Illustrating the Pathology of Secretion of Pus in the Liver

**Published:** 1852-02

**Authors:** 


*Case illustrating the Pathology of Secretion of Pus in the Liver.* Reported by JOHN A. SWETT, M. D., Physician to the New York Hospital. *Suppurative Portal Phlebitis.**

A gentleman, fifty-eight years of age, had generally enjoyed good health, except that, fifteen years ago, he had an affection of his stomach, which was suspected, at the time, to be of a cancerous nature, but from which he perfectly recovered by visiting the Sulphur Springs of Virginia. During the eighteen months, also, preceding the fatal sickness, he had noticed, occasionally, a slight tendency to diarrhœa, which did not interrupt, however, his usual good health. Indeed his friends, just before his last sickness, regarded his health as unusually good. During the early part of the autumn, he had been somewhat exposed for two or three weeks to the malarious influence.

On the 10th of October, he attended to his business as usual during the morning; but in the afternoon, he returned home, feeling unwell. In the evening he sent for his physician to see him. He found him complaining of a muddy feeling across the forehead, of nausea with slight vomiting of bilious matter, and of slight colic-like pain in the abdomen. His skin was rather feverish, and his pulse about one hundred in a minute. He took some purgative medicine, containing blue pill, which operated well, producing healthy, natural stools, and affording relief.

The next day, Oct. 11th, he continued to be slightly feverish; his pulse was still accelerated, and his skin began to assume a yellowish tinge. He complained of a sensation of inward fever, as he called it, which he referred to the epigastric region. About this time, also, he began to experience chills.

The jaundice continued to increase, and the chills returned irregularly, sometimes three or four times in a day, followed by increased heat, which soon terminated in profuse perspiration. There was no complaint of pain, nor of tenderness on pressure anywhere, and especially over the region of the liver, which was carefully examined. The cerebral functions, as well as those of the chest were quite undisturbed. The pulse was generally below one hundred in a minute. There was some appetite, the patient enjoying a little chicken soup and tea with cracker. The bowels were kept regular by simple purgatives and by injections.

About the 22d of October, two weeks after his illness commenced, the

---

* This report presents the history of a case of a rare form of disease, the diagnosis of which must be attended frequently, if not generally, with considerable difficulty. The case is also interesting as illustrative of the general symptoms associated with suppurative phlebitis.—EDITOR BUFFALO JOURNAL.

chills assuming more regularity and the remission of the fever seeming to be more distinct, and there being no local pain or uneasiness, indeed no evidence of any local affection except the jaundice, and with this, the stools were quite natural in appearance, ten grains of the sulphate of quinine were administered, and afterward five grains every four hours, until three doses had been taken. This treatment was at first followed by marked relief. The next day he took five grains every four hours, until he had taken three doses. The third day he took but two doses, as the improvement first experienced from the use of the remedy did not continue, and the chills had returned. He also complained of deafness, and of ringing in the ears.

A temporizing plan of treatment was now pursued again. The patient used effervescing draughts, and took with some relief a bowl of chicken soup. The bowels were regulated by simple means; a little sulphuric acid, and afterward the nitro-muriatic acid, which the patient enjoyed very much, was prescribed, and a foot bath of the same was used at night. There was still no complaint of pain in the region of the liver, and firm pressure, especially upward under the ribs, in the right hypochondriac region, and percussion in the same region discovered no tenderness. The pulse continued to range at about one hundred in a minute. But the chills returned irregularly, followed by increased fever, and rapidly by profuse perspiration. The yellowness of the skin was diminishing. About the 28th of October, the febrile symptoms having again assumed a more regular type, quinine was again prescribed, but with no benefit. It was, therefore, omitted, and the expectant plan of treatment again adopted.

This plan of treatment was continued until the 3d of November. The patient, when asked how he felt, invariably answered, "Perfectly comfortable." There was still no pain or tenderness on pressure over the region of the liver. The jaundice was diminishing. The pulse was about one hundred in a minute, soft and regular. The patient slept tolerably at night. His tongue was slightly coated, but he took nourishment with a certain relish. His intellect was perfectly clear, and he was now quiet and composed. The chills had again assumed more regularity. Every afternoon, at about half-past 3 o'clock, a chill would occur, followed by fever, and rapidly by profuse perspiration; but, during the night, the face was flushed and the skin hot and dry. Towards the morning, the excitement seemed to abate, and the pulse became less frequent, and from this time until three or four o'clock in the afternoon, the patient was more comfortable. Still, a slight degree of chilliness would recur, irregularly, at different periods of the day. It was determined to try again the effect of quinine. A grain every hour, for three successive hours, was prescribed, when the chill recurred at the usual hour, 3 o'clock in the afternoon. It was administered again in the same doses, commencing at 2 P. M., of the 4th of November, when the paroxysm of the preceding afternoon had subsided. It was continued through the day, and at 3 o'clock in the afternoon, a full dose of laudanum was administered. The chill did not recur, nor ever afterward.

The quinine was omitted as soon as the chill had ceased, on the morning of the 6th of November; and the case was again treated by the expectant plan. A little brandy was allowed, and beef tea was directed to be given.

On the evening of Nov. 6th, there was some vomiting; the abdomen was becoming tympanitic, and then the prostration, which had been great, became more decided. Still, there was no abdominal pain or tenderness, and there were two natural stools following a simple injection. The tendency to sinking continued during the night, and on the morning of Nov. 7th, the patient was decidedly worse. His pulse was feeble and irregular, his skin was inclined to be cold, his features were altered, and he vomited at times. Still, his intellect was perfectly clear, his manner composed and tranquil, and he experienced no abdominal pain or tenderness, although the tympanitis continued.

During the day, with the use of moderate stimulation, he rallied gradually. His pulse became regular, had improved in strength and had fallen to 104 in a minute. The temperature of the skin was quite natural. There was no tendency to chill, or to perspiration. Once during the afternoon, he complained of pain in the right side of the abdomen, which was relieved by pressure.

The succeeding night was passed comfortably. The patient slept a good deal, awaking at intervals, and on the morning of Nov. 9th, he was apparently more comfortable than the day before. His pulse had fallen to ninety-six in a minute, and was regular, but rather feeble. There was no febrile excitement. The tongue was moist and but slightly coated. The stomach was tranquil and there was no complaint of pain. The patient gave his often repeated answer, "I am perfectly comfortable." But about the middle of the day, the mind began to wander a little, and a tendency to restlessness ensued, with hiccough. A severe attack of abdominal pain ensued, which was relieved by a mustard plaster. The abdomen was still distended, and it felt very hard on pressure, which, however, produced no decided pain. During the evening the patient gradually sank, and died during the night.

*On post mortem examination*, the evidences of general peritonitis existed. Lymph and sero-purulent matter was effused in the peritonial cavity. There was but little lymph effused on the surface of the liver, or of the spleen, and none upon the surface of the stomach. The liver was not enlarged. It was of a dark and rather greenish hue, and somewhat flabby. The portal vein was hard and firm, and when examined was found filled with lymph, partly firm, partly broken down into a grumous detritus. The branches of this vein as they ramified through the liver, were filled with healthy pus. The hepatic veins were not affected. The substance of the liver was also quite healthy The mesenteric vein supplying the small intestines was also filled with pus, and the mesentery itself was much thickened, and its cellular tissue contained numerous collections of pus. The stomach and the intestinal canal, examined as low as the rectum, were quite healthy, except that the coats were somewhat thickened. The other abdominal organs were also healthy. The lung contained a few cretaceous tubercles. The brain was not examined. *N. Y. Medical Times.*

